# Co-expression of LASS2 and TGF-β1 predicts poor prognosis in hepatocellular carcinoma

**DOI:** 10.1038/srep32421

**Published:** 2016-09-01

**Authors:** Haoyu Ruan, Ting Wang, Chen Yang, Guangzhi Jin, Dishui Gu, Xuan Deng, Cun Wang, Wenxin Qin, Haojie Jin

**Affiliations:** 1 State Key Laboratory of Oncogenes and Related Genes, Shanghai Cancer Institute, Renji Hospital, Shanghai Jiao Tong University School of Medicine, Shanghai, China; 2 Department of Pathology, Eastern Hepatobiliary Surgery Hospital, Second Military Medical University, Shanghai, China.

## Abstract

Longevity assurance homolog 2 of yeast LAG1 (LASS2) has been reported to act as an important tumor suppressor in the development of human cancers. However, little is known about the prognostic value of LASS2 in hepatocellular carcinoma (HCC) . In the present study, we analyzed correlation between LASS2 and TGF-β1 levels, and evaluated their prognostic values in HCC patients. We first analyzed the expression of LASS2 and TGF-β1 in two independent cohorts (test cohort: 184 HCC patients; validation cohort: 118 HCC patients) using immunohistochemistry (IHC). Kaplan-Meier survival and Cox regression analyses were executed to evaluate the prognosis of HCC. The results of IHC analysis revealed a positive correlation between the expression of LASS2 and TGF-β1. HCC Patients with low expression of LASS2 and TGF-β1 had shorter overall survival (OS) and time to recurrence (TTR) than patients with high expression of LASS2 and TGF-β1. Furthermore, combination of LASS2 and TGF-β1 was an independent and significant risk factor for OS and TTR. In conclusion, low expression of LASS2 and TGF-β1 contributes to the aggressiveness and poor prognosis of HCC, and may represent a novel prognostic biomarker for HCC patients.

Hepatocellular carcinoma (HCC) is one of the most common malignant tumors, the third major cause of cancer-related death worldwide^1^. Despite many improvements have been achieved in clinical treatments such as liver surgical resection, chemotherapy and transplantation, HCC patients still have high recurrence incidence and poor prognosis^2^,^3^. The prognosis of HCC patients after surgical resection remains unsatisfactory, with a 5-year survival rate ranged from 25–39%, and a 5-year recurrence rate ranged from 65–80%^4^,^5^,^6^. Therefore, it is critical to identify valuable factors for prognosis prediction and novel therapeutic strategies.

Homo sapiens longevity assurance homolog 2 of yeast LAG1 (LASS2), which is also known as tumor metastasis suppressor gene (TMSG1) or ceramide synthase 2 (CerS2), is the gene identified from a human liver cDNA library by our laboratory^7^. As one member of LASS family, including LASS1–6, LASS2 mRNA is at the highest level of all LASS members, and has the broadest tissue distribution, particularly abundant in the liver, kidney and brain in mice^8^. A growing amount of research has reported that LASS2 mainly acts as a tumor suppressor and is closely associated with a variety of tumor progression, including prostate^9^, breast^10^, bladder^11^ and liver cancer^12^. Our previous investigations have shown that low expression of LASS2 is associated with poor prognosis in patients with breast cancer^10^, and deletion of LASS2 is associated with a high risk of spontaneous or diethylnitrosamine (DEN)-induced HCC in hepatocyte-specific LASS2-knockout (KO) mice models^13^,^14^. However, no study has been performed to assess whether there are any correlations among the protein level of LASS2, clinicopathological parameters, and the survival rate in clinical HCC samples.

Transforming growth factor-β1 (TGF-β1) plays biphasic functions in tumor tumorigenesis, having a growth inhibitory effect at early stages, but at later stages enhancing the malignant conversion. The TGF-β1 signaling could cross-talk with multiple intracellular signaling in tumor cell. Generally, TGF-β1 signal is transduced from plasma membrane to the nucleus via serine/threonine kinase receptor type I (TβR-I) and type II (TβR-II) and their downstream effectors, smads. Moreover, TGF-β1 signal is also transduced via Smad-independent pathways through activation of mitogen-activated protein kinase (MAPK), nuclear factor kappa-B (NF-κB) and phosphatidylinositol 3-kinase (PI3K)^15^. As a multifunctional cytokine, TGF-β1 regulates important cellular processes including cell growth, differentiation, apoptosis, epithelial-mesenchymal transition (EMT), immunosuppressant and senescence^16^,^17^,^18^,^19^. Lots of evidences show that TGF-β1 functions as either a tumor suppressor or a tumor promoter mainly depending on the stage of carcinogenesis^20^,^21^,^22^. TGF-β1 particularly achieves its tumor suppressive effect by inhibiting cell cycle progression through G1-arrest^23^,^24^, and inducing apoptosis^25^. Besides, TGF-β1 can also induce senescence of epithelial cells and HCC^18^,^26^,^27^,^28^. In a previous study, we found that LASS2 was closely associated with TGF-β1 levels in DEN-induced liver tumorigenesis^13^. In the present study, we investigated the relationships between LASS2 and TGF-β1, evaluated their prognostic values in two independent cohorts of HCC patients, as well as their relationships with clinicopathological parameters.

## Results

### The relationship between LASS2 and TGF-β1 in HCC patients

**Figure 1:**
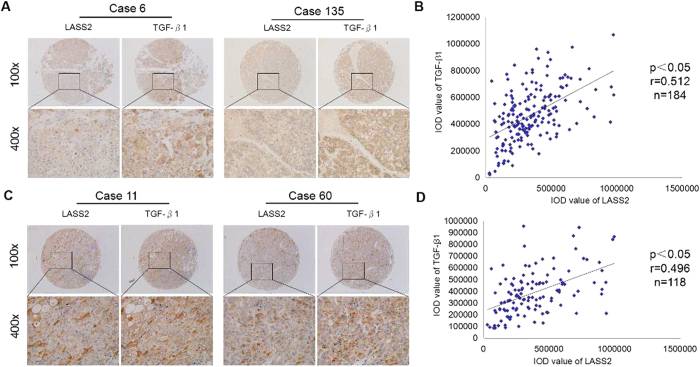
The relationship between LASS2 and TGF-β1 in HCC patients. (**A**). Two representative cases of LASS2 and TGF-β1 expression in the test cohort. (**B**). Relevance of LASS2 and TGF-β1 in the test cohort. (Pearson’s correlation, r = 0.512, n = 184, p < 0.05). (**C**). Two representative cases of LASS2 and TGF-β1 expression in the validation cohort. (**D**). Relevance of LASS2 and TGF-β1 in the validation cohort. (Pearson’s correlation, r = 0.496, n = 118, p < 0.05).

We enrolled a test cohort consisting of 184 HCC patients and a validation cohort of 118 HCC patients, who had undergone curative resection between 2008 and 2013. Using immunohistochemical staining, we examined expression of LASS2 and TGF-β1 in the two independent cohorts, and analyzed their coexpression. The observed LASS2 staining pattern in HCC tissues was both plasma membranous and cytoplasmic. The level and extent of staining varied from weak focal to extensive strong expression. For TGF-β1 staining, TGF-β1 was primarily localized in the cytoplasm of tumor cells, which were producing TGF-β1 (Fig. 1A,C). Based on analysis of integrated optical density (IOD) value, we found that a significant positive correlation between expression of LASS2 and TGF-β1 was observed in the test cohort (Pearson’s correlation, r = 0.512, n = 184, p < 0.05, Fig. 1B). Similarly, the positive correlation between LASS2 and TGF-β1 was also observed in the validation cohort (Pearson’s correlation, r = 0.496, n = 118, p < 0.05, Fig. 1D).

### Low expression of LASS2 and TGF-β1 significantly correlated with progression and poor prognosis of HCC in the test cohort

Among the 184 HCC patients of test cohort, 64 (34.8%) and 100 (54.3%) cases had high expression of LASS2 and TGF-β1, respectively. We performed further analyses to determine the clinicopathological significance of LASS2 and TGF-β1 in HCC. The expression level of LASS2 was negatively correlated with the tumor size, tumor differentiation, and TNM stage (Table 1). In addition, we also found that TGF-β1 expression was correlated with age and TNM stage (Table 1).

**Figure 2:**
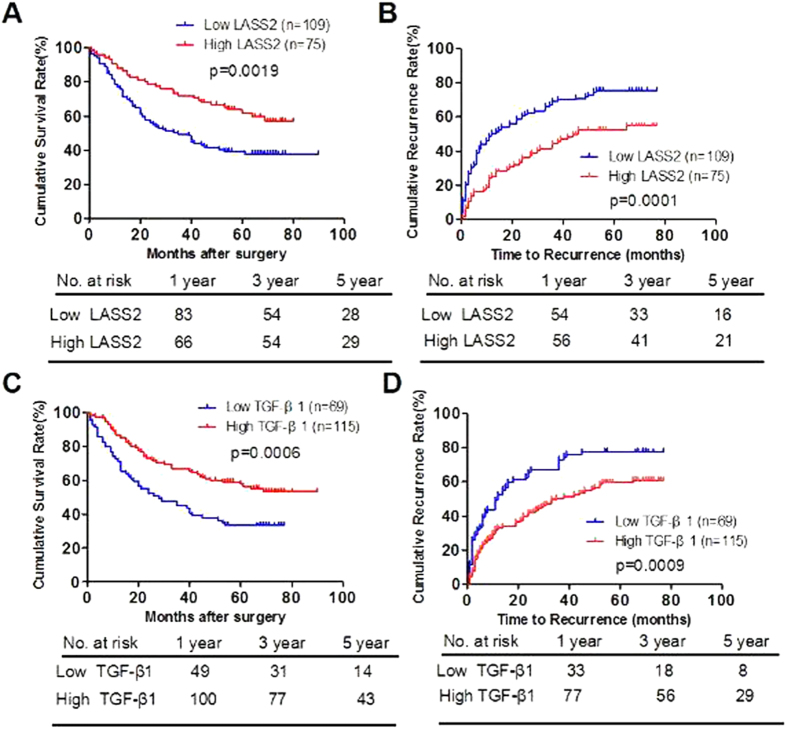
Low expression of LASS2 and TGF-β1 significantly correlates with poor prognosis of hepatocelluar carcinoma in the test cohort. (**A**,**B**). Kaplan-Meier analysis of the correlation between LASS2 expression and OS (**A**) or TTR (**B**) in the test cohort. (**C**,**D**). Kaplan-Meier analysis of the correlation between TGF-β1 expression and OS (**C**) or TTR (**D**) in the test cohort. Log-rank tests were used to determine statistical significance.

We determine the prognostic value of LASS2 and TGF-β1 in the test cohort of HCC patients. OS and TTR were evaluated using the Kaplan-Meier method. Our results showed that the median OS time was 46.5 (95% CI: 39.7–53.3) months for HCC patients with low expression of LASS2 and 58.4 (95% CI: 51.9–64.9) months for HCC patients with high expression of LASS2 (p =  0.0019, Fig. 2A). The median TTR was 28.9 (95% CI: 23.2–34.5) months for patients with HCC who had low expression of LASS2 and 45.9 (95% CI: 38.9–53.0) months for patients with HCC who had high expression of LASS2 (p = 0.0001, Fig. 2B). Accordingly, we found that HCC patients with low expression of TGF-β1 had shorter OS (38.1 versus 60.5 months, p = 0.0006, Fig. 2C) and TTR (26.5 versus 41.4 moths, p = 0.0009, Fig. 2D).

### The combination of LASS2 and TGF-β1 exhibited the improved prognostic value for HCC patients in the test cohort

**Figure 3:**
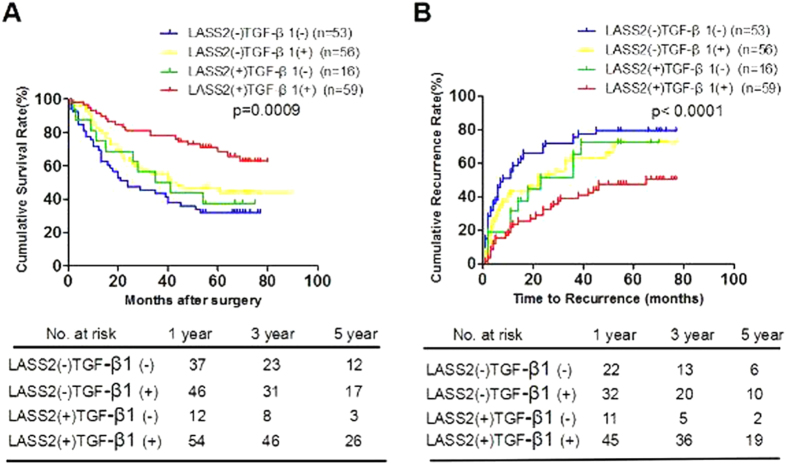
The combination of LASS2 and TGF-β1 exhibited the improved prognostic value for HCC patients in the test cohort. (**A**,**B**). Kaplan-Meier analysis of the correlation between LASS2/TGF-β1 and OS (**A**) or TTR (**B**) in the test cohort. GI, LASS2−/TGF-β1−(n = 53); GII, LASS2−/TGF-β1 + (n = 56); GIII, LASS2+/TGF-β1-(n = 16); GIV, LASS2+/TGF-β1 + (n = 59).

To analyze the prognostic value of combining LASS2 and TGF-β1 levels for HCC in the test cohort, we divided patients into the following four groups, such as: low LASS2 low TGF-β1 group, high LASS2 low TGF-β1 group, low LASS2 high TGF-β1 group, and high LASS2 high TGF-β1 group. Using the Kaplan–Meier method, low LASS2 low TGF-β1 group had the shortest OS (median months: 36.6, 95% CI: 28.5–44.8) and TTR (median months: 23.9, 95% CI: 16.1–31.7), whereas high LASS2 high TGF-β1 group had the longest OS (median months: 62.4, 95% CI: 55.5–69.2) and TTR (median months: 48.9, 95% CI: 41.0–56.7) (Fig. 3). Collecting, the results indicated that the combination of low LASS2 expression and low TGF-β1 expression in HCC tissues appeared to be predictive of the poorest prognosis.

### Univariate and multivariate analyses of prognostic variables for HCC patients in the test cohort

To evaluate whether the expression levels of LASS2 and TGF-β1 were independent prognostic value for HCC patients in the test cohort, univariate and multivariate analyses using a Cox regression model were applied. As shown in Table 2, LASS2 level, TGF-β1 level, and the LASS2/TGF-β1 combination was responsible for the OS and TTR of HCC patients. In addition to the above factors, serum AFP, TNM stage, child-pugh, tumor size, tumor number, microvascular invasion, and tumor differentiation were also correlated with OS and/or TTR (Table 2). Factors showed significance in univariate analysis were then subjected to multivariate Cox proportional hazards analysis. Despite neither LASS2 nor TGF-β1 alone were independent prognostic markers for OS and TTR, the LASS2/TGF-β1 combination was an independent prognostic marker for both OS and TTR in the test cohort. These results indicated that the LASS2/TGF-β1 combination had a potent prognostic value compared with LASS2 or TGF-β1 alone.

### Confirmation of prognostic ability in an independent validation cohort

**Figure 4:**
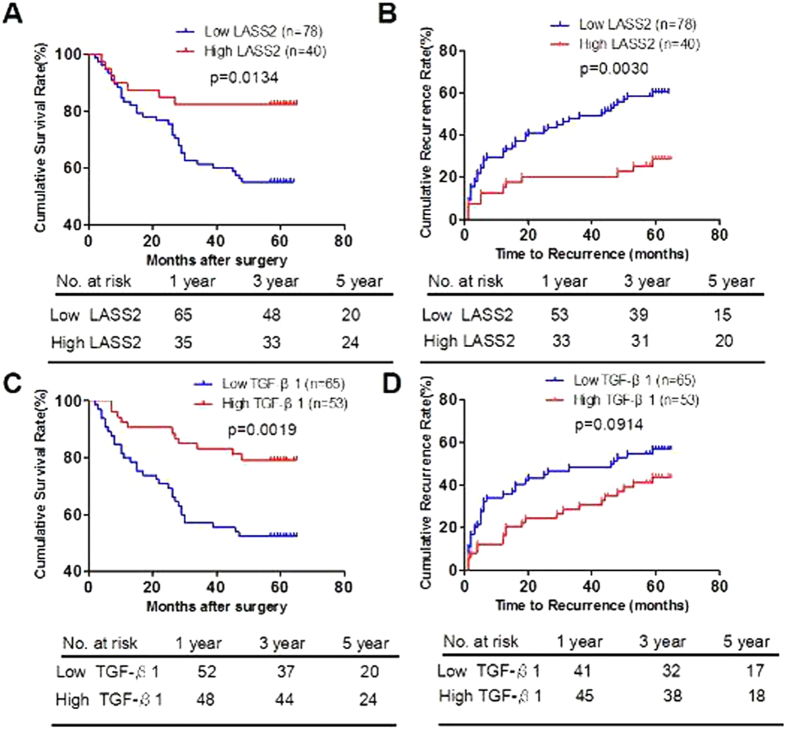
Low expression of LASS2 and TGF-β1 significantly correlates with poor prognosis of hepatocelluar carcinoma in the validation cohort. (**A**,**B**). Kaplan-Meier analysis of the correlation between LASS2 expression and OS (**A**) or TTR (**B**) in the validation cohort. (**C**,**D**). Kaplan-Meier analysis of the correlation between TGF-β1 expression and OS (**C**) or TTR (**D**) in the validation cohort. Log-rank tests were used to determine statistical significance.

We observed similar prognostic results of LASS2 and TGF-β1 in another independent cohort consisting of 114 HCC patients after surgery. Among the 114 HCC patients of validation cohort, 40 (35.1%) and 53 (46.5%) cases had high expression of LASS2 and TGF-β1, respectively. Kaplan–Meier analysis showed that low LASS2 group had the shorter OS (median months: 44.7, 95% CI: 39.6–49.9) and TTR (median months: 36.1, 95% CI: 30.2–42.0), whereas high LASS2 group had the longrer OS (median months: 55.8, 95% CI: 49.4–62.1) and TTR (median months: 52.4, 95% CI: 45.1–59.6) (Fig. 4A,B).

**Figure 5:**
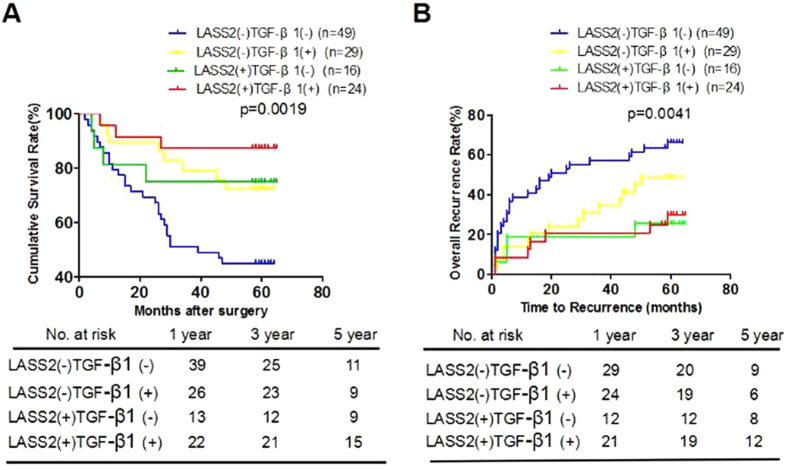
The combination of LASS2 and TGF-β1 exhibited the improved prognostic value for HCC patients in the validation cohort. (**A**,**B**). Kaplan-Meier analysis of the correlation between LASS2/TGF-β1 and OS (**A**) or TTR (**B**) in the validation cohort. GI, LASS2−/TGF-β1−(n = 49); GII, LASS2−/TGF-β1 + (n = 29); GIII, LASS2+/TGF-β1-(n = 16); GIV, LASS2+/TGF-β1 + (n = 24).

Furthermore, we found that patients with low TGF-β1 expression also had shorter OS (42.7 versus 56.2 months, p =  0.0019, Fig. 4C) and TTR (36.4 versus 48.5 months, p =  0.0914, Fig. 4D). Importantly, low LASS2 low TGF-β1 group had the shortest OS (median months: 39.5, 95% CI: 32.8–46.3) and TTR (median months: 31.2, 95% CI: 23.5–38.8), whereas high LASS2 high TGF-β1 group had the longest OS (median months: 58.8, 95% CI: 52.1–65.5) and TTR (median months: 52.5, 95% CI: 43.4–61.6) (Fig. 5). LASS2/TGF-β1 combination was associated with OS (p = 0.001) and TTR (p = 0.001) by univariate analysis (Table 2) and was an independent predictor for both OS (p = 0.009) and TTR (p = 0.025) by multivariate analysis (Table 3).

## Discussion

Hepatocellular carcinoma is one of the most aggressive cancers featured with high rate of metastasis and mortality. Although several molecular biomarkers have been reported to have clinical significance for predicting HCC prognosis^29^,^30^,^31^, clinical and molecular features to be used as prognostic parameters in clinical practice are still substantially needed. Critical oncogenes or tumor suppressors involved in different stages of growth, cell cycle progression, disease initiation and responses to environmental stimuli provide important clues to identify prognostic biomarkers of cancer. LASS2 is the gene identified from a human liver cDNA library by our laboratory and interacts with proteolipid subunit of vacuolar H^+^ ATPase (VPL)^7^. A growing amount of research has reported that LASS2 mainly acts as a tumor suppressor and is closely associated with a variety of tumor progression, including prostate^9^, breast^10^, bladder^11^ and liver cancer^12^. LASS2 can induce apoptosis of prostate cancer, inhibit growth and invasion of breast cancer cell *in vitro* through interacting with vacuolar ATPase^32^,^33^. Our previous studies have shown that low expression of LASS2 is associated with poor prognosis in patients with breast cancer^10^, and deletion of LASS2 is associated with a high risk of spontaneous or DEN-induced HCC in hepatocyte-specific LASS2-KO mice models^13^,^14^. Recent research also revealed that LASS2 was the target of miR-9, and involved in the chemoresistance of bladder cancer^11^. In addition, it has been reported that decreased expression of LASS2 is associated with worse prognosis in meningiomas^34^. However, the prognostic significance of LASS2 expression in HCC is still unclear. In current study, we found that the expression of LASS2 level was negatively correlated with the tumor size, tumor differentiation, and TNM stage. Moreover, the Kaplan-Meier survival analysis showed that the OS and TTR of HCC patients with low LASS2 expression were shorter than those with high LASS2 expression. These results suggested that LASS2 may act as a critical tumor suppressor in HCC progression.

Our previous study showed that knockout of LASS2 significantly upregulated the expression of TGF-β1 in DEN-induced liver tumourigenesis of mice^13^. This clue strongly prompted us to further study the correlation between LASS2 and TGF-β1 in HCC patients. Interestingly, we were surprised to find that a significant positive correlation between expression of LASS2 and TGF-β1 was observed in HCC patients. This discrepancy may be mainly explained by the following points: 1) In the process of cancer development, TGF-β1 plays biphasic functions in tumor tumorigenesis, having a growth inhibitory effect at early stages, but at later stages enhancing the malignant conversion. This contradictory results may be due to the function switch of TGF-β1 in different stages of carcinogenesis, which is still largely unclear since now. 2) Although the animal models of DEN-induced HCC were used as a tool to test preventive treatments for HCC, the mice model of DEN-induced hepatocarcinogenesis and the actual process of human liver cancer are still different. For example, our study was performed mainly on hepatitis B virus (HBV)-related HCC, which is a dominant etiology of Asian HCC patients. According to the statistics, 157 of 184 (85.3%) HCC patients had positve hepatitis B surface antigen (HBsAg) in the test cohorts of this study, and 99 of 114 (86.8%) HCC patients had positve HBsAg in our validation cohort. 3) The correlation between LASS2 and TGF-β1 was firstly observed in a hepatocyte-specific LASS2-KO transgenic mouse model, which was generated using the Cre/LoxP system for site-specific excisional DNA recombination. The particular mouse model also might lead to its inconsistent with clinical results. However, the underlying mechanism remains to be further investigated.

TGF-β1 particularly achieves its tumor suppressive effect through the inhibition of proliferation and the induction of apoptosis or senescense in the early stage-tumors^18^,^19^. Consistent with a tumor-suppressor role for TGF-β1, analysis of clinical samples and tumor-derived cell lines suggested that it is a common occurrence that reduction in epithelial responsiveness to TGF-β1 during carcinogenic progression in many tissues^35^. It was reported that TGF-β1 can induce p53-independent and p16-independent, and reactive oxygen species (ROS)-dependent senescence arrest in well-differentiated HCC cells^18^. In our study, most HCC patients of our study were well and moderate differentiation. According to the statistics, 182 of 184 (98.9%) and 114 of 114 (100%) HCC patients were well and moderate differentiation in the test cohort and validation cohort, respectively. The Kaplan-Meier survival analysis also showed that the OS and TTR of HCC patients with low TGF-β1 expression were shorter than those with high TGF-β1 expression.

In the present study, we provided the evidence that high level of LASS2/TGF-β1 expression predicted a favorable OS and TTR rate for HCC patients. The 1-, 3- and 5-year OS rates of HCC patients with high level of LASS2 or TGF-β1 expression were remarkably higher than those of HCC patients with low levels of LASS2 or TGF-β1 expression, respectively. Accordingly, the 1-, 3- and 5-year TTR rates of HCC patients with high level of LASS2 or TGF-β1 expression were significantly lower than those of HCC patients with low levels of LASS2 or TGF-β1 expression, respectively. As combination of multiple markers might yield more information for predicting clinical outcome of HCC patients, combination of LASS2 and TGF-β1 expression were therefore used as a predictor of clinical outcome. In both test cohort and validation cohort, HCC patients with both high expression of LASS2 and TGF-β1 had the most favorable OS and TTR rates, whereas those patients with both low expression of LASS2 and TGF-β1 had the poorest OS and TTR rates. Besides, we used univariate and multivariate analyses to further study the prognostic value of LASS2 and TGF-β1, the results showed that combination of LASS2 and TGF-β1 was significant and independent prognostic factor for HCC patients, but either LASS2 or TGF-β1 was not.

In conclusion, our study demonstrated that high levels of LASS2 expression and TGF-β1 expression have a positive relationship with prognosis of HCC patients. The combination of LASS2 and TGF-β1 was more sensitive than either of them alone with respect to OS and TTR, and could be used as a new independent prognostic marker of HCC patients.

## Materials and Methods

### Patients and tissue samples

This study involved two independent cohorts of patients with HCC: test cohort (184 HCC patients) and validation cohort (118 HCC patients), obtained from Eastern Hepatobiliary Surgery Hospital (Shanghai, PR China) between January 2003 and April 2006. For suspected cases with elevated serum AFP level (>20 ng/ml as positive), computed tomography (CT) and/or magnetic resonance imaging (MRI) were used to verify tumor recurrence. No patient received either radiotherapy or chemotherapy before the surgery, and no other cancers co-occurrence. The overall survival (OS) was defined as the length of time between the surgery and death or the last follow-up examination. The time to recurrence (TTR) was calculated from the date of tumor resection until the detection of tumor recurrence, death or the last observation. Tumor stage was defined according to the American Joint Committee on Cancer (AJCC 2010, 7th edition) TNM staging system^36^. The grade of tumor differentiation was assigned by the Edmondson-Steiner grading system. Micrometastases were defined as tumors adjacent to the border of the main tumor that was only observed under the microscope^37^.

For the use of clinical materials for research purposes, prior patients’ consents and approval were obtained from the Ethics Committee of Renji Hospital, Shanghai Jiao Tong University School of Medicine and EHBH of the Second Military Medical University. Written informed consent was obtained from all subjects for publication of this study. All experiments were performed in accordance with approved guidelines of Shanghai Jiao Tong University School of Medicine.

### Immunohistochemistry and Scoring

HCC tumor tissues were resected from the patients, and then were fixed with 10% formalin and embedded in paraffin before prepared for tissue microarray. Briefly, 1-mm cores were taken from intratumoral tissue and serial sections (4-μm thick) were placed on slides coated with 3-aminopropyltriethoxysilane. The immunohistochemistry analysis was conducted as described previously^30^. Immunostaining scores were independently evaluated by two pathologists who were blinded to the clinical outcome. The sections were incubated with primary goat anti-human LASS2 polyclonal antibody (clone sc-65102, 1:80, Santa Cruz Biotechnology)^38^ and rabbit anti-human TGF-β1 polyclonal antibody (18978-1-AP, 1:100, Proteintech)^39^ at 4 °C overnight, and then secondary antibody within 30 minutes. The average sum of integrated optical density (IOD) of each sample was calculated using ImageJ software.

### Statistical Analysis

Differences among variables were assessed by χ^2^ analysis or two-tailed Student *t* test. Kaplan-Meier analysis was used to assess survival. Log-rank tests were used to compare survival of patients between subgroups. Multivariate analyses were performed by multivariate Cox proportional hazard regression model. Data were presented as mean ± SEM. Differences were considered to be statistically significant for p < 0.05.

## Additional Information

**How to cite this article**: Ruan, H. *et al*. Co-expression of LASS2 and TGF-β1 predicts poor prognosis in hepatocellular carcinoma. *Sci. Rep*. **6**, 32421; doi: 10.1038/srep32421 (2016).

## Figures and Tables

**Table 1:** Relationships of LASS2 and TGFβ1 expression level with the clinicopathological features in HCC patients from test and validation cohorts.

Characteristics	Test					*p-value*	Validation					*p-value*
	LASS2		*p*-value	TGF-β1			LASS2		p-value	TGF-β1		
	Low (n = 109)	*High* (*n* = *75*)		Low (n = 69)	*High* (*n* = *115*)		Low (n = 78)	*High* (*n* = *40*)		Low (n = 65)	*High* (*n* = *53*)	
Sex			0.862			0.585			0.129			0.549
male	94	64		58	100		73	34		58	49	
female	15	11		11	15		5	6		7	4	
Age			0.173			**0**.009*			0.810			0.705
≤50	62	35		45	52		33	16		28	21	
>50	47	40		24	63		45	24		37	32	
HBsAg			0.673						0.767			0.460
negative	15	12		9	18	0.628	12	7		9	10	
positive	94	63		60	97		66	33		56	43	
Serum AFP			0.637			0.282			0.927			0.560
≤20 ng/ml	37	28		21	44		26	13		20	19	
>20 ng/ml	72	47		48	71		52	27		45	34	
Liver cirrhosis			0.127			0.966			0.337			0.898
no	36	17		20	33		28	18		25	21	
yes	73	58		49	82		50	22		40	32	
TNM stage			0.545			0.193			**0**.030*			**0**.030*
I	32	27		21	38		24	18		18	24	
II	61	40		35	66		39	21		34	26	
III-IV	16	8		13	11		15	1		13	3	
Child-pugh			0.301			0.366			0.965			0.395
A	102	67		65	104		70	36		57	49	
B	7	8		4	11		8	4		8	4	
Tumor size			**0**.003*			0.109			0.614			0.131
≤3 cm	16	25		11	30		20	12		14	18	
>3 cm	93	50		58	85		58	28		51	35	
Tumor number (multiple vs single)			0.635			0.460			0.074			0.101
single	84	60		52	92		57	35		47	45	
multiple	25	15		17	23		21	5		18	8	
Tumor differentiation			**0**.033*			0.538			0.487			0.978
well	6	13		5	14		5	4		5	4	
moderate	102	61		63	100		73	36		60	49	
poor	1	1		1	1		0	0		0	0	
Microvascular invasion			0.512			0.691			0.087			0.083
no	37	29		26	40		30	22		24	28	
yes	72	46		43	75		48	18		41	25	

Statistical analyses were done by the Chi-square (χ2) test.

^*^p-value smaller than 0.05.

**Table 2:** Univariate analyses of factors associated with recurrence and survival in hepatocelluar carcinoma patients from test and validation cohorts.

Variables	Test				Validation			
	OS		TTR		OS		TTR	
	HR (95% CI)	*p-value*	HR (95% CI)	*p-value*	HR (95% CI)	*p-value*	HR (95% CI)	*p-value*
Sex (male vs female)	1.558(0.911–2.664)	0.105	1.298(0.786–2.142)	0.308	0.659(0.204–2.132)	0.486	0.612(0.221–1.690)	0.343
Age (>50 vs ≤50)	1.004(0.674–1.495)	0.984	1.067(0.748–1.523)	0.719	1.418(0.746–2.695)	0.286	1.365(0.792–2.354)	0.262
HBsAg (positive vs negative)	1.400(0.765–2.565)	0.275	1.256(0.752–2.098)	0.384	1.504(0.591–3.826)	0.392	1.840(0.789–4.291)	0.158
Serum AFP (>20 vs ≤20)	2.928(1.787–4.797)	**<0**.001*	1.890(1.278–2.794)	**0**.001*	1.252(0.651–2.408)	0.501	1.635(0.906–2.951)	0.103
Liver cirrhosis (yes vs no)	1.144(0.726–1.804)	0.562	1.192(0.796–1.786)	0.394	2.107(1.059–4.195)	**0**.034*	3.127(1.651–5.921)	**<0**.001*
TNM stage (I *vs* II *vs* III–IV)	1.569(1.144–2.152)	**0**.005*	1.813(1.352–2.432)	**<0**.001*	2.877(1.766–4.687)	**<0**.001*	2.405(1.587–3.644)	**<0**.001*
Child-pugh (A vs B)	1.881(1.004–3.527)	**0**.049*	2.080(1.167–3.708)	**0**.013*	1.233(0.485–3.138)	0.660	0.997(0.428–2.324)	0.994
Tumor size (>3 cm vs ≤3 cm)	2.682(1.464–4.912)	**0**.001*	1.691(1.073–2.665)	**0**.024*	1.876(0.868–4.055)	0.110	2.504(1.227–5.108)	**0**.012*
Tumor number (multiple vs single)	1.721(1.103–2.686)	**0**.017*	2.279(1.533–3.388)	**<0**.001*	4.550(2.464–8.405)	**<0**.001*	3.038(1.748–5.283)	**<0**.001*
Tumor differentiation (well vs moderate vs poor)	1.856(0.939–3.670)	0.075	1.841(1.031–3.288)	**0**.039*	1.982(0.479–8.202)	0.345	2.826(0.689–11.595)	0.149
Microvascular invasion (yes vs no)	1.495(0.971–2.301)	0.068	1.515(1.034–2.219)	**0**.033*	1.574(0.837–2.960)	0.159	1.500(0.875–2.570)	0.140
LASS2 (low vs high)	0.514(0.334–0.791)	**0**.002*	0.517(0.354–0.756)	**0**.001*	0.347(0.154–0.781)	**0**.011*	0.367(0.190–0.710)	**0**.003*
TGF-β1 (low vs high)	0.507(0.340–0.757)	**0**.001*	0.577(0.403–0.828)	**0**.003*	0.354(0.178–0.705)	**0**.003*	0.581(0.339–0.996)	**0**.049*
LASS2 and TGF-β1†GI vs GII vs GIII vs GIV	0.716(0.601–0.852)	**<0**.001*	0.733(0.629–0.855)	**<0**.001*	0.558(0.397–0.786)	**0**.001*	0.640(0.492–0.833)	**0**.001*

Univariate analysis: Cox proportional hazards regression model.

Validation cohort: GI, LASS2−/TGF-β1−(n = 49); GII, LASS2−/TGF-β1 + (n = 29); GIII, LASS2+/TGF-β1-(n = 16); GIV, LASS2+/TGF-β1 + (n = 24).

HbsAg: Hepatitis B surface antigen; HR: Hazard ratio; OS: Overall survival; TTR: Time to recurrence.

^*^p-value smaller than 0.05.

^†^Test cohort: GI, LASS2−/TGF-β1−(n = 53); GII, LASS2−/TGF-β1 + (n = 56); GIII, LASS2+/TGF-β1-(n = 16); GIV, LASS2+/TGF-β1 + (n = 59).

**Table 3:** Multivariate analyses of factors associated with survival and recurrence in hepatocelluar carcinoma patients from test and validation cohorts.

Variables	OS		TTR	
	HR (95% CI)	*p-value*	HR (95% CI)	*p-value*
Test cohort				
Serum AFP (>20 vs ≤20)	2.860(1.742–4.693)	<0.001	1.879(1.265–2.790)	0.002
TNM stage (I *vs*I I *vs* III–IV)	NA	NA	NA	NA
Child-pugh (A vs B)	NA	NA	2.438(1.353–4.392)	0.003
Tumor size (>3 cm vs ≤3 cm)	2.191(1.183–4.059)	0.013	NA	NA
Tumor number (multiple vs single)	1.763(1.127–2.759)	0.013	2.493(1.666–3.731)	<0.001
Tumor differentiation (well vs moderate vs poor)	NA	NA	NA	NA
Microvascular invasion (yes vs no)	NA	NA	NA	NA
LASS2 (low vs high)	NA	NA	NA	NA
TGF-β1 (low vs high)	NA	NA	NA	NA
LASS2 and TGF-β1^†^				
GI vs GII vs GIII vs GIV	0.753(0.631–0.898)	0.002	0.705(0.603–0.824)	<0.001
Validation cohort				
Liver cirrhosis (yes vs no)	2.179(1.087–4.369)	0.028	3.755(1.958–7.200)	<0.001
TNM stage (I *vs*I I *vs* III–IV)	NA	NA	1.896(1.234–2.914)	0.004
Tumor size (>3 cm vs ≤3 cm)	NA	NA	2.719(1.304–5.671)	0.008
Tumor number (multiple vs single)	4.036(2.149–7.580)	<0.001	NA	NA
LASS2 (low vs high)	NA	NA	NA	NA
TGF-β1 (low vs high)	NA	NA	NA	NA
LASS2 and TGF-β1^†^				
GI vs GII vs GIII vs GIV	0.629(0.444–0.891)	0.009	0.732(0.557–0.962)	0.025

Multivariate analysis: Cox proportional hazards regression model. Variables were adopted for their prognostic significance by univariate analysis.

HbeAg: Hepatitis B e-antigen; HR: Hazard ratio; NA: Not applicable; OS: Overall survival; TTR: Time to recurrence.
